# Anesthetic Management in a Pregnant Woman With Super Morbid Obesity and Severe Obstructive Sleep Apnea Syndrome Undergoing Cesarean Section: A Case Report

**DOI:** 10.7759/cureus.90128

**Published:** 2025-08-15

**Authors:** Takayuki Morimoto, Hiroyuki Ogino, Kentaro Hara, Michiko Yamaguchi, Tetsuya Hara

**Affiliations:** 1 Department of Anesthesiology and Intensive Care Medicine, Nagasaki University Graduate School of Biomedical Sciences, Nagasaki, JPN; 2 Department of Anesthesiology, National Hospital Organization Nagasaki Medical Center, Nagasaki, JPN; 3 Healthcare Management Research Center, Chiba University Hospital, Chiba, JPN; 4 Department of Operation Center, National Hospital Organization Nagasaki Medical Center, Nagasaki, JPN

**Keywords:** bilevel positive airway pressure (bipap), combined spinal-epidural anesthesia (csea), noninvasive positive pressure ventilation (nppv), obesity, ramped position (ramp), sleep apnea syndrome

## Abstract

Pregnant women with super morbid obesity (Class III obesity) and severe obstructive sleep apnea syndrome (SAS) undergoing cesarean section present significant anesthetic challenges. Although the ramped position (RAMP) and noninvasive positive pressure ventilation (NPPV) may optimize respiratory function, their intraoperative use in this population is not widely reported. A 36-year-old pregnant woman with obesity (BMI = 50 kg/m²) and severe SAS underwent cesarean section under combined spinal-epidural anesthesia. RAMP and bilevel positive airway pressure (BiPAP) were employed to optimize respiratory function. Despite achieving a T4 sensory blockade, the prolonged duration of surgery resulted in upper abdominal pain, necessitating propofol sedation. This led to respiratory depression, which was successfully managed by adjusting the BiPAP settings. RAMP and NPPV, particularly BiPAP, may be beneficial in optimizing respiratory management during cesarean section in pregnant women with super morbid obesity and severe SAS, especially in cases requiring unexpected sedation.

## Introduction

Obesity in pregnant women is associated with an increased risk of comorbidities like hypertension, diabetes, fetal abnormalities, higher cesarean section rates, and labor-related complications [1, 2]. In particular, pregnant women with obesity and obstructive sleep apnea syndrome (SAS) face significantly higher risks of mortality [3]. Cesarean section in this population presents substantial perioperative challenges, including intraoperative respiratory impairment and a tendency toward prolonged operative time and postoperative hospitalization, necessitating careful anesthetic planning [4, 5]. Given these concerns, respiratory management in pregnant women with super morbid obesity and severe SAS undergoing cesarean sections under neuraxial anesthesia requires special attention. However, limited literature discusses the intraoperative use of the ramped position (RAMP) and noninvasive positive pressure ventilation (NPPV) in such cases.

Herein, we present a case in which a combination of RAMP and NPPV, specifically bilevel positive airway pressure (BiPAP), was successfully employed for safe anesthetic management in a pregnant woman with super morbid obesity (BMI = 50 kg/m²) and severe SAS undergoing cesarean section. Noninvasive respiratory support in continuous positive airway pressure (CPAP) mode was instituted at the onset of neuraxial anesthesia; after unplanned sedation precipitated respiratory depression, the settings were adjusted to BiPAP spontaneous/timed (S/T) mode, maintaining adequate ventilation and oxygenation. These observations offer insights into optimizing respiratory management in similar high-risk cases.

## Case presentation

The patient was a 36-year-old multiparous woman (gravida 6, para 2) with a BMI of 50 kg/m² (height: 164.7 cm, weight: 135.5 kg; pre-pregnancy BMI: 46.4 kg/m²; Class III obesity). She had a prior cesarean section six years earlier owing to fetal macrosomia, with a repeat procedure planned for this pregnancy, including tubal ligation for contraception.

She had severe SAS with an apnea-hypopnea index of 100, but had discontinued CPAP therapy. Her baseline peripheral oxygen saturation in room air was in the low 90s, and neither pulmonary function tests nor arterial blood gas analysis were performed during this or her previous pregnancy. She was unable to tolerate a supine position owing to respiratory distress. Preoperative evaluation revealed cardiomegaly (cardiothoracic ratio on chest radiography, 58.9%) and mild pulmonary hypertension (tricuspid regurgitation pressure gradient on transthoracic echocardiography, 31 mmHg), likely secondary to pregnancy-related cardiac load and hypoxemia. Left ventricular function was preserved (ejection fraction: 64%), with no significant cardiac dysfunction.

Anesthetic management

Given her severe obesity, combined spinal-epidural anesthesia (CSEA) was anticipated to be technically challenging. However, owing to the high risk of airway management difficulties and the need to avoid neonatal respiratory depression, CSEA was chosen over general anesthesia. The patient had previously undergone CSEA successfully for her prior cesarean section, further supporting this decision. Nevertheless, her weight had increased by 10 kg since the last surgery, raising concerns regarding procedural difficulties. Consequently, we were also prepared for airway management with a supraglottic airway and a videolaryngoscope for a potential conversion to general anesthesia.

**Figure 1:**
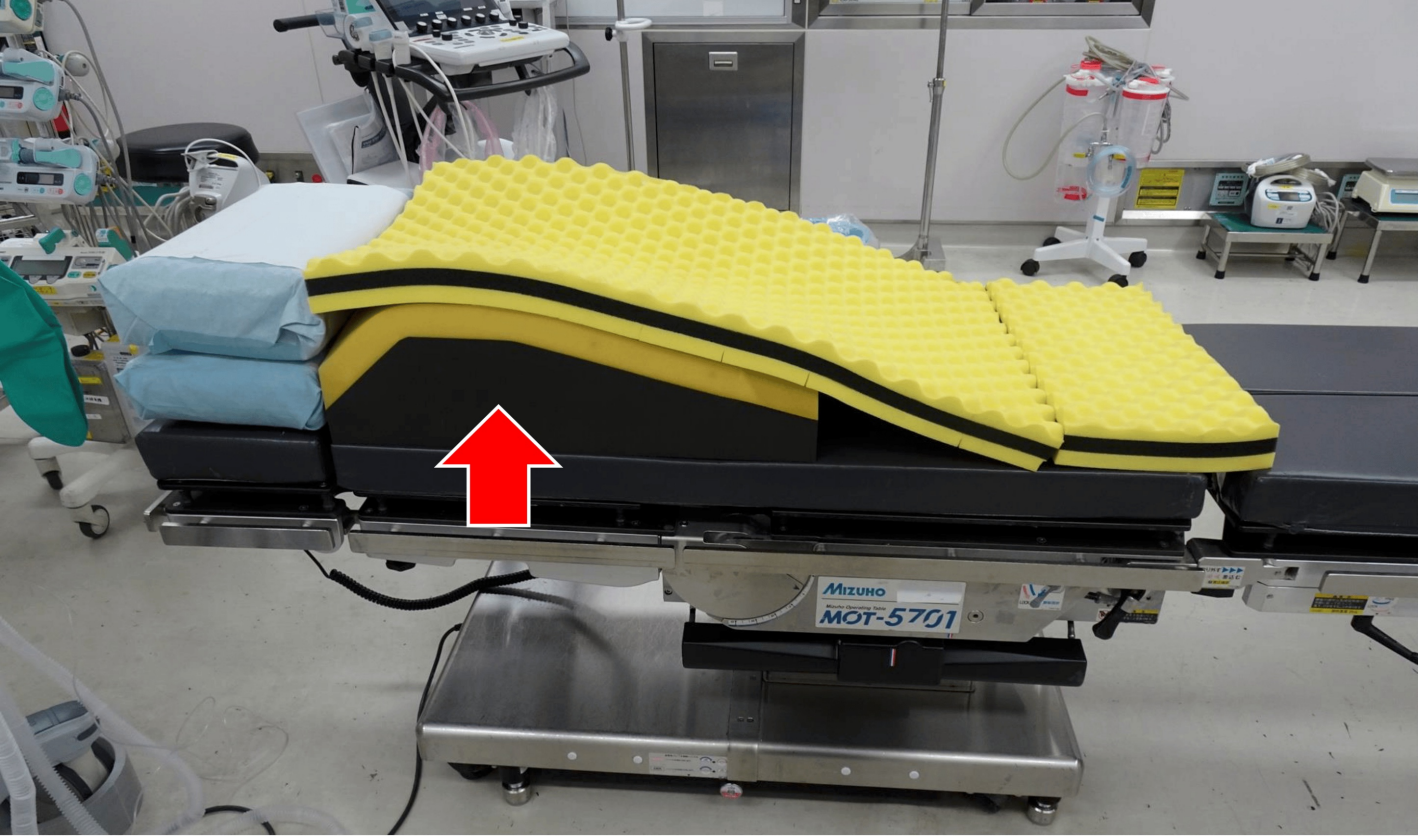
Pillows used in RAMP The Purefix SRxⅡ Prone cushion (red arrow; Hopes, Tokyo, Japan) and standard operating room cushions were used to establish RAMP. RAMP: ramped position

Owing to her severe SAS, the patient could not tolerate a supine position, necessitating intraoperative positioning using the RAMP technique (head-up tilt of approximately 20°). Initially, only CPAP was considered; however, due to the risk of respiratory muscle suppression from cephalad anesthetic spread, BiPAP (NKV-330; Nihon Kohden, Tokyo, Japan) was chosen instead. A preoperative simulation in RAMP confirmed that surgery could proceed without significant respiratory distress. The Purefix SRxⅡ Prone cushion (Hopes, Tokyo, Japan) and standard operating room cushions were used to establish RAMP (Figure 1).

Intraoperative course

On the day of surgery, she adhered to the preoperative fasting guidelines. An ultrasound-guided pre-scan in the seated position identified intervertebral spaces before CSEA. An epidural catheter was inserted at the L1/2 interspace (depth: 12 cm subcutaneously, 5 cm intrathecally). Spinal anesthesia was administered at the L3/4 interspace, with cerebrospinal fluid return at 8 cm, followed by 0.5% hyperbaric bupivacaine 2.2 mL (11 mg) with fentanyl 20 μg administration.

**Figure 2:**
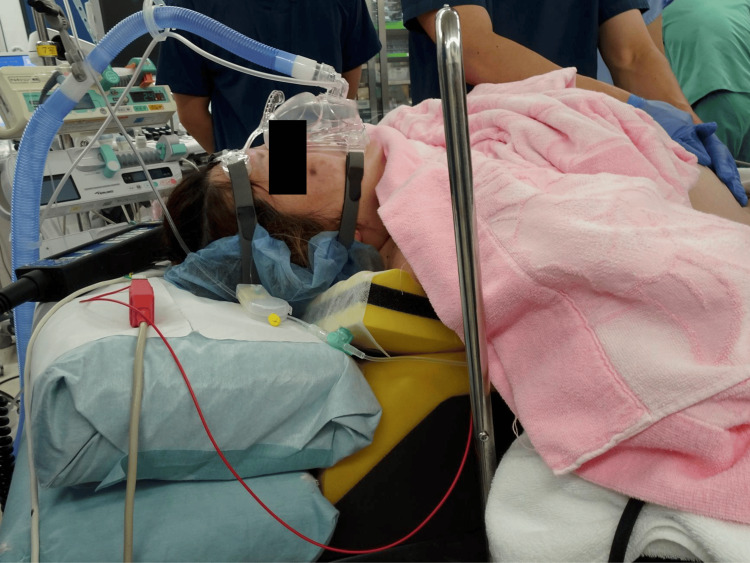
RAMP and BiPAP attachment after anesthesia induction BiPAP was initiated in CPAP mode following anesthetic induction. RAMP: ramped position, BiPAP: bilevel positive airway pressure, CPAP: continuous positive airway pressure

Immediately after spinal anesthesia, the patient was positioned in RAMP, and BiPAP was initiated in CPAP mode with a positive end-expiratory pressure of 5 cmH₂O and a fraction of inspired oxygen (FiO_2_) of 0.4 (Figure 2). She reported no respiratory discomfort.

**Figure 3:**
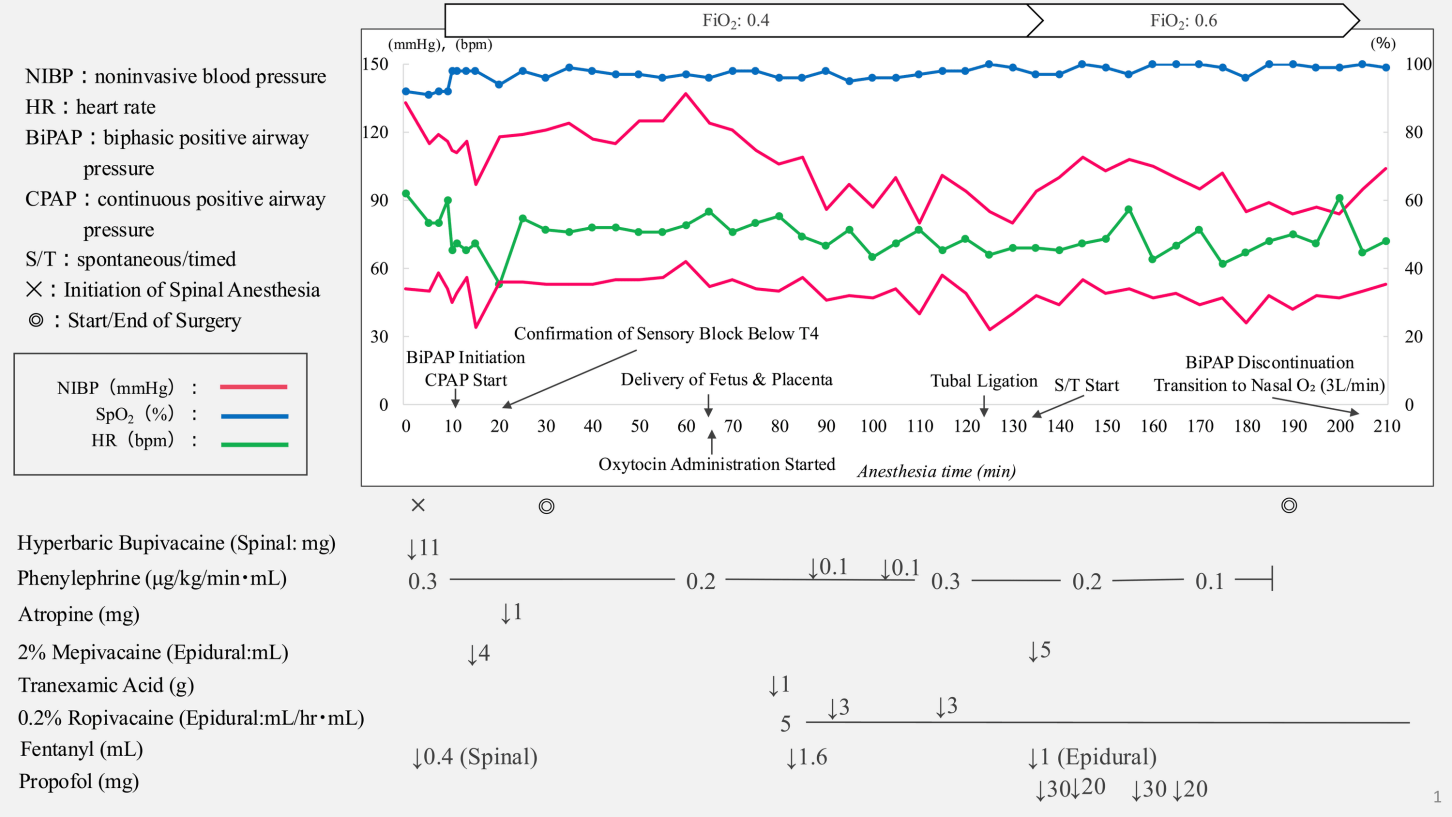
Anesthesia record from the start of anesthesia SpO_2_, NIBP, and HR measurements, along with major drug administration, events, and their progression, are documented. SpO_2_: peripheral oxygen saturation, NIBP: noninvasive blood pressure, HR: heart rate, FiO_2_: fraction of inspired oxygen, BiPAP: bilevel positive airway pressure, CPAP: continuous positive airway pressure, S/T: spontaneous/timed

The anesthesia records are shown in Figure 3. Ten minutes following spinal anesthesia, sensory blockade remained at T10, prompting epidural administration of mepivacaine (80 mg) to extend the block below T4, enabling surgical initiation. Given her severe obesity and surgical history, fetal delivery required 1 h post-anesthesia induction. During surgery, mild epigastric pain was managed with continuous epidural infusion of 0.2% ropivacaine at 5 mL/h (10 mg/h) supplemented by intermittent 3 mL (6 mg) boluses. However, approximately 2 h post-anesthesia induction, abdominal lavage following tubal ligation triggered intolerable epigastric pain, causing movement. Mild sedation with propofol (20-30 mg boluses) was administered, but respiratory depression occurred. BiPAP was switched to S/T mode with inspiratory and expiratory positive airway pressure of 7 and 5 cmH₂O, respectively, and an FiO_2_ of 0.6, maintaining adequate ventilation and oxygenation without complications. 

Postoperative course

After surgery and before ward transfer, the patient was fully awake, and BiPAP was discontinued. Oxygenation remained stable on nasal oxygen (3 L/min), and CPAP therapy was resumed after transfer to the ward. The final sensory blockade level was at T6. The total anesthesia duration and surgical duration were 187 min and 153 min, respectively. The newborn had an Apgar score of 9 at both 1 and 5 min, with no significant abnormalities.

Postoperatively, respiratory and circulatory statuses remained stable, and pain was managed via epidural 0.2% ropivacaine (5 mL/h, 10 mg/h) and intravenous acetaminophen (1,000 mg every 6 h).

The mother and child were discharged on postoperative day 5.

## Discussion

This is a valuable case demonstrating successful anesthetic management of a pregnant woman with super morbid obesity and severe obstructive SAS undergoing cesarean section using RAMP and NPPV. There have been previous reports of using RAMP and BiPAP in combination [6], but this is the first report of the effectiveness of BiPAP when unexpected sedation was required.

RAMP aligns the external auditory meatus with the sternum, optimizing the oral and laryngeal axes [7], improving lung compliance, functional residual capacity, and oxygenation, which are often compromised by obesity and pregnancy [8]. Additionally, RAMP is useful for securing the field of view during intubation when converting to general anesthesia [8]. A previous report demonstrated successful oxygenation and intubation in an obese pregnant woman (BMI = 59 kg/m²) undergoing cesarean section with RAMP under general anesthesia [9]. However, its head-up tilt may limit cephalad spread of hyperbaric bupivacaine, potentially compromising sensory blockade. Therefore, a multimodal approach should incorporate CSEA over spinal anesthesia alone, epidural volume extension, consideration of a higher thoracolumbar epidural puncture level, and appropriate adjustments to the selection of drugs and dosages in neuraxial anesthesia.

Beyond positioning strategies, optimizing respiratory support is essential in obese pregnant women with severe SAS. NPPV, including CPAP and BiPAP, improves oxygenation, ventilation, and respiratory mechanics. A prior study reported that intraoperative CPAP use in obese patients undergoing spinal anesthesia for lower limb and lower abdominal surgeries improved respiratory function [10]. In a previous study reporting a case of uterine surgery in a patient with obesity and SAS managed with CSEA and propofol sedation, the use of BiPAP resulted in good oxygenation and avoidance of intubation [11]. However, considering the possibility that BiPAP alone may not respond well to central respiratory depression due to sedation, it is necessary to prepare for emergency airway management, such as a supraglottic airway and a videolaryngoscope.

Another concern is the potential aspiration risk of using NPPV intraoperatively in pregnant women with morbid obesity without a secured airway. In this case, the risk was mitigated by strict adherence to preoperative fasting guidelines and the head-up tilt position by RAMP. A systematic review of intraoperative NPPV in 618 patients (including six cesarean sections) reported no aspiration incidents and concluded that intraoperative NPPV is feasible and safe in appropriately selected patients [12]. Therefore, the use of NPPV in pregnant women with morbid obesity should be limited to elective surgeries in which preoperative fasting guidelines can be strictly followed.

In this case, RAMP and NPPV alleviated respiratory distress, ensuring stable surgical conditions. BiPAP in spontaneous/timed mode was also able to respond to respiratory depression caused by sudden sedation. Thus, RAMP and NPPV, especially BiPAP, may be important options in the anesthetic management of a pregnant woman with super morbid obesity and severe obstructive SAS undergoing cesarean. However, it is possible that sedation would not have been necessary in this case if sufficient analgesia had been provided. This may have been due to the fact that the operation time for cesarean section tends to be prolonged in pregnant women with super morbid obesity, and the head-up tilt with RAMP may limit cephalad spread of hyperbaric bupivacaine. This means that when using RAMP, a strategy of neuraxial anesthesia must be developed for adequate analgesia.

In general, CSEA is preferred over spinal anesthesia alone for managing pregnant women with morbid obesity, as it provides better control in prolonged surgeries and minimizes hypotension risk by reducing the intrathecal bupivacaine dose [1]. However, in obese patients, epidural catheter failure rates are two to three times higher [13], potentially limiting effectiveness and necessitating precise spinal anesthesia planning.

Some studies suggest that the intrathecal bupivacaine dose required for a T6 sensory level is comparable between obese and non-obese pregnant women [14], with no significant correlation between BMI, body weight, and sensory blockade when a fixed bupivacaine dose (12 mg) is used [15]. Therefore, dose reduction based solely on obesity is not recommended. On the other hand, bupivacaine dosage directly influences anesthesia duration [16], with the 95% effective dose for hyperbaric bupivacaine in cesarean section ranging from 8.8 to 15 mg [17]. In this case, 11 mg of hyperbaric bupivacaine with fentanyl was administered, which may not have been a particularly low dose. However, considering the potential for limited cephalad spread due to RAMP and the development of upper abdominal pain later in the procedure, a higher dose might have prolonged anesthesia more effectively.

As for opioids added to spinal anesthesia, fentanyl alone provided insufficient analgesia, and intrathecal morphine could have offered longer pain relief [18]. Alternatively, combining fentanyl and morphine might have ensured sustained analgesia, though careful monitoring for postoperative nausea, vomiting, and acute spinal opioid tolerance would be necessary [19].

As for the baricity of bupivacaine, the isobaric bupivacaine provides longer analgesia in lower limb surgeries [20]. However, a systematic review of cesarean sections found no significant differences between isobaric and hyperbaric bupivacaine in terms of additional analgesia requirements or conversion to general anesthesia [21]. Thus, baricity may not significantly impact analgesia duration in abdominal surgeries. However, RAMP’s head-up tilt may redistribute hyperbaric bupivacaine caudally, reducing block height. Isobaric bupivacaine could be a viable alternative, requiring further study to refine neuraxial anesthesia strategies in obese pregnant women with severe SAS undergoing cesarean section with RAMP.

## Conclusions

For pregnant women with super morbid obesity and severe SAS, RAMP and NPPV effectively optimize perioperative respiratory function, reducing ventilation and oxygenation risks. Given the potential for unplanned sedation and the risk of respiratory muscle suppression from cephalad anesthetic spread, BiPAP should be readily available for respiratory support. 
